# Vesicle protrusion induced by antimicrobial peptides suggests common carpet mechanism for short antimicrobial peptides

**DOI:** 10.1038/s41598-024-60601-w

**Published:** 2024-04-27

**Authors:** Peter Park, Danilo K. Matsubara, Domenico R. Barzotto, Filipe S. Lima, Hernan Chaimovich, Siewert J. Marrink, Iolanda M. Cuccovia

**Affiliations:** 1 Departamento de Bioquímica, Instituto de Química, Universidade de São Paulo, São Paulo, Brazil; 2 Departamento de Química Fundamental, Centro de Ciências Exatas e da Natureza, Universidade Federal de Pernambuco, Recife, Brazil; 3 Groningen Biomolecular Sciences and Biotechnology Institute (GBB), University of Groningen, 9747 AG Groningen, the Netherlands

**Keywords:** Biochemistry, Biophysics, Computational biology and bioinformatics, Surface chemistry

## Abstract

Short-cationic alpha-helical antimicrobial peptides (SCHAMPs) are promising candidates to combat the growing global threat of antimicrobial resistance. They are short-sequenced, selective against bacteria, and have rapid action by destroying membranes. A full understanding of their mechanism of action will provide key information to design more potent and selective SCHAMPs. Molecular Dynamics (MD) simulations are invaluable tools that provide detailed insights into the peptide-membrane interaction at the atomic- and meso-scale level. We use atomistic and coarse-grained MD to look into the exact steps that four promising SCHAMPs—BP100, Decoralin, Neurokinin-1, and Temporin L—take when they interact with membranes. Following experimental set-ups, we explored the effects of SCHAMPs on anionic membranes and vesicles at multiple peptide concentrations. Our results showed all four peptides shared similar binding steps, initially binding to the membrane through electrostatic interactions and then flipping on their axes, dehydrating, and inserting their hydrophobic moieties into the membrane core. At higher concentrations, fully alpha-helical peptides induced membrane budding and protrusions. Our results suggest the carpet mode of action is fit for the description of SCHAMPs lysis activity and discuss the importance of large hydrophobic residues in SCHAMPs design and activity.

## Introduction

In recent years, antimicrobial resistance (AMR) has become a growing global health concern^1–3^. A 2022 systematic global analysis revealed 4.95 million deaths were associated with bacterial antibiotic resistance^4^. It has been estimated that antibiotic-resistant diseases will kill as many as 10 million people per year by 2050, which is more than the number of people who die from cancer worldwide^5^.

The strategies to combat AMR can be categorized on two fronts: prevention and treatment. Some of the strategies in the prevention category are public awareness, access to sanitation, increasing vaccine coverage, reducing in the misuse of antibiotics in agriculture^5,6^ and clinical practices^7^, and rapid diagnostics^3^. On the treatment front, the development of novel antibiotics and the improvement of currently used drugs are the main challenges^2–4^.

One category of antimicrobials that has shown promising results is antimicrobial peptides (AMPs)^8^. These are found in several different organisms across all kingdoms of life as part of their innate defense system and mainly kill bacteria by disrupting their cell membranes^2,6,8^. Short cationic alpha-helical antimicrobial peptides (SCHAMPs) are short sequenced AMPs rich in basic and hydrophobic aminoacids, which characteristically adopt a random conformation in water and an amphipathic alpha-helical structure in nonpolar solvents, and negatively charged bilayers and micelles^8,9^.

SCHAMPs display selectivity towards bacterial membranes compared to mammalian cells due to the higher content of negatively charged lipids^8,10^ and absence of sterols^11,12^ and are also effective against fungi^8,13^, biofilms^14^, and even cancer cells, as demonstrated by Decoralin^15^. Their shorter size offers several advantages such a lesser cost of production in bulk and the fact that their its composition can be easily tunable in case of requirements regarding toxicity, stability, or half-life^16^.

Despite their promising attributes, their use of SCHAMPs has been limited due to a lack of detailed understanding of their mechanism of action on membranes. Current models for membrane disruption by AMPs include pore-formation, i.e., peptides forming a transmembrane pore either by themselves or together with lipids, and the carpet mechanism, which postulates a more surfactant-like behavior with peptides adsorbing at the membrane/water interface, leading to membrane destabilization beyond a critical peptide-to-lipid (P/L) ratio^9^. Due to their shorter length, the formation of pores by SCHAMPs is less likely^17–20^, and increasing evidence favors the carpet mechanism^21–25^. Using KIAGKIA (Lys-Ile-Ala-Gly-Lys-Ile-Ala) motifs of varied lengths, transmembrane pores form only when AMPs are long enough to span the hydrophobic bilayer core^19^.

A detailed molecular description of the SCHAMPs carpet mechanism is still to be investigated, as such understanding is a fundamental step in designing more efficient and selective AMPs. Molecular Dynamics (MD) simulations are invaluable computational tools to study molecular systems in atomistic detail and have been widely used to investigate the interaction between AMPs and membranes at the molecular level^26–28^.

Atomistic simulations that successfully describe AMP binding and pore formation show that these processes happen in microseconds^29,30^. Because of this, coarse-grained models are commonly used to describe how different AMPs, like maculatin 1.1^31^, alamethicin^32^, magainin-2^29,33–35^, and melittin^34^, form pores. Most of the AMPS that have been studied are long (> 17 residues) and can pass through the hydrophobic thickness of synthetic lipid bilayers^19^. Because of this, it is important to be careful when putting SCHAMPs in the same category as larger AMPs in terms of how they work.

Our earlier atomistic MD simulations of SCHAMP BP100 (KKLFKKILKYL), a mix of AMPs cecropin A and melittin^36^, showed that the negatively charged membranes’ alpha-helical structure helps the dehydration of BP100's hydrophobic part by rotating the peptide and inserting it into the membrane core^37^. We coined the term “peptide flip” to describe such dynamical process wherein peptides transition from a solvated state into a membrane-bound state. In addition, single BP100 causes local membrane thinning and negative curvature and slows lipid lateral diffusion^38^. The peptide binds better to the liquid-disordered phase than the liquid-ordered phase in In coarse-grained MD simulations of BP100 on a phase-separated membrane. It also has a low tendency to oligomerize, even at high concentrations^35^.

To investigate the occurrence of a common mechanism of action for SCHAMPs, we selected BP100 and three other peptides. The criteria for peptide selection were the similarity in the number of aminoacids, a random conformation in an aqueous solution, and an alpha-helical structure in the presence of negatively charged membranes. We chose Neurokinin-1 (RPKPQQFFGLM)^39,40^ (also known as Substance P), a human neurotransmitter with AMP properties; Decoralin (SLLSLIRKLIT)^41^, a SCHAMP isolated from the venom of *Oreumenes decorates* wasps, and Temporin-L^42^, from the skin of the frog *Rana temporaria,* which displayed the highest activity among temporins*.* The folding behavior of all four peptides has been extensively studied through both experimental^21,36,40,41,43,44^ and theoretical approaches^37,45–47^.

In this present study, we used atomistic MD simulations of BP100, Decoralin, NK-1, and Temporin-L on anionic membrane models to explore the occurrence of a common initial binding step and its effect on local membrane properties. We further explored the effect of peptide concentration on vesicles using coarse-grained MD, analyzing vesicle structural alterations. Together, our results support a carpet-like mechanism for these peptides, which may extend to the wide-range of available SCHAMPs and provide key information for designing more efficient, selective and less cytotoxic antibiotics.

## Methods

### Peptides

**Figure 1:**
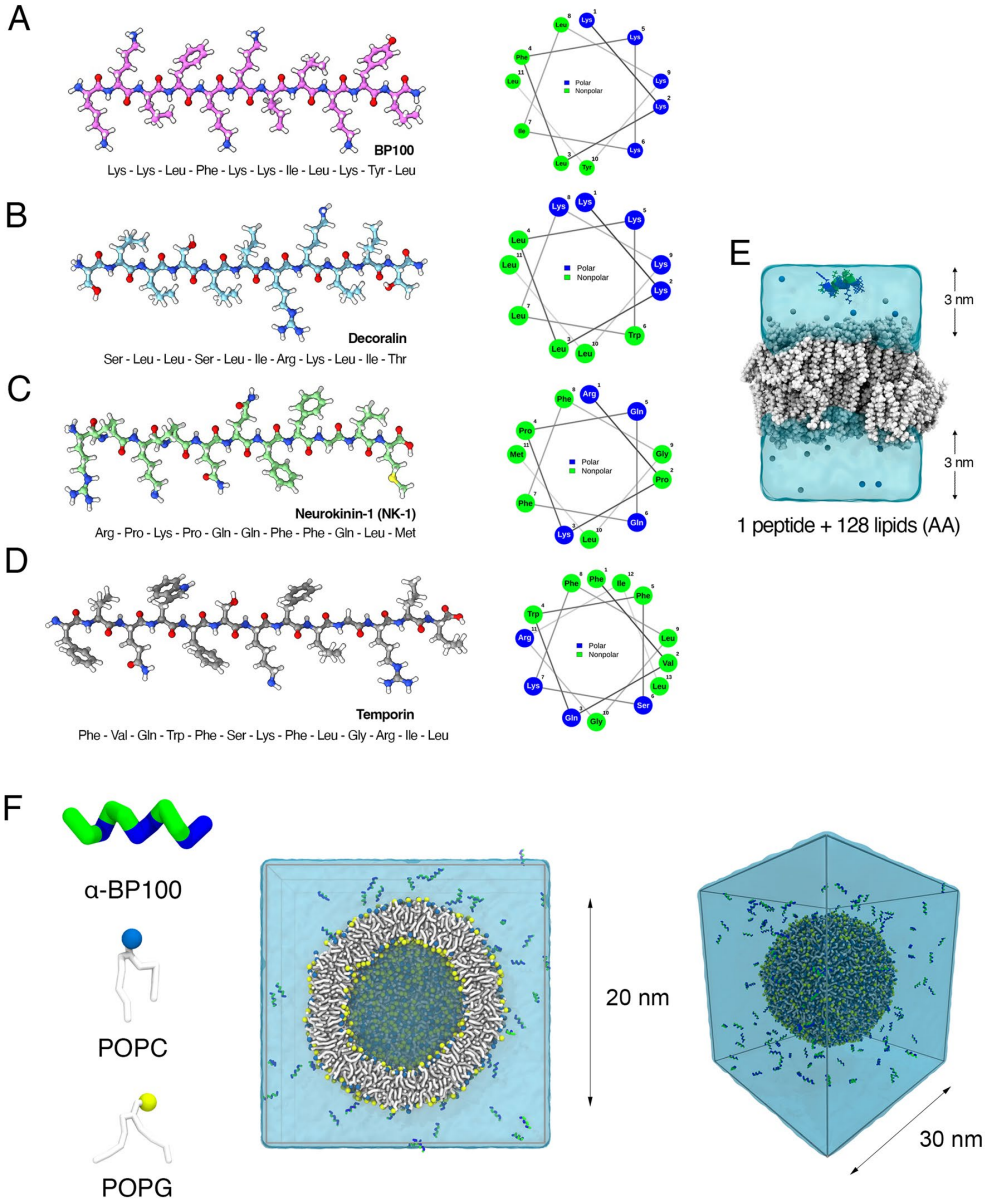
Antimicrobial peptides and set-ups used in this study. BP100 (**A**), Decoralin (**B**), NK-1 (**C**), and Temporin-L (**D**) atomic structures and their respective helical wheel projections. (**E**) All-atom membrane system setup with a single peptide placed in solution. (F) CG representation of BP100 and POPC/POPG lipids, together with the vesicle setup (left image: cross section, right image: entire box view) in which multiple peptides were inserted in the extra-vesicular solution. Images rendered in VMD 1.9.4^57,58^ and UCSF ChimeraX.

Three SCHAMPs (Fig. 1B–D) were selected from the Antimicrobial Peptide Database^48^ (APD3, https://aps.unmc.edu) based on their similarity with a previously studied SCHAMP, BP100 (Fig. 1A)^37,38^. They are short peptides (11–13 aminoacids), amphiphilic in alpha-helix conformation, which is their predominant secondary structure when in contact with negatively charged membranes^21,36,40,41,43,44^. And in solution, as BP100, they have no secondary structure.

### Molecular dynamics

All simulations were run with GROMACS 5.1.4^49–51^ version and analyzed with GROMACS 2020.6.

#### All-atom simulations set-ups and analysis

For all-atom simulations, the *ff99sb-ildn-NMR*^52^ force field was used for the peptides and the SLipids^53–55^ force field for the lipids. Peptides were initially folded as alpha-helices without constraints and positioned approximately 2 nm away from the membrane (Fig. 1E). Previous data suggest negatively charged membranes favor the occurrence of peptide flip^37^, in which the amphiphilic peptide approaches the membrane with its polar side and then it turns, facing the membrane with its non-polar side and burying the peptide into the membrane core. In order to compare with previous results obtained with BP100^37,38^, we simulated peptides on a mixed bilayer of palmitoyloleoylphosphatidylcholine (POPC) and palmitoyloleoylphosphatidylglycerol (POPG) in a 1:1 mol/mol ratio. Membranes containing 64 lipids on each leaflet were assembled using PACKMOL^56^ and were solvated with the TIP3P water model, with an average of 55 water molecules per lipid. After sodium counter-ions were added to neutralize the systems, all set-ups were equilibrated. Membrane-only and peptide-only systems were also simulated and used as controls.

Simulations were run with a time-step of 2 fs and all bonds were constrained using the LINCS^59^ algorithm. Neighbor-searching was accomplished using the Verlet cut-off algorithm at every 50 fs. Short-range electrostatic and van der Waals interactions were computed using a 1.5 nm cut-off, using a potential-switch function from a 1.4 nm distance. Long-range electrostatic interactions were treated using the Particle Mesh Ewald^60^ method. Temperature was set to 323 K, above the lipid transition temperature, and coupled using the V-rescale^61^ thermostat with a coupling constant of 0.1 ps. Semiisotropic pressure was coupled using the Parrinello-Rahman barostat^62^ at 1 bar with a coupling constant of 1.0 ps. Periodic boundary conditions were applied in all three dimensions. Energy minimization step was performed using the steepest descent method, followed by NVT and NPT steps. Each production run was carried out for 2 μs for each of the four types of peptide.

Peptide hydration and lipid clustering analysis were calculated using *gmx trjorder,* computing the number of waters and lipids surrounding the peptides along the simulation, using a 0.5 and 0.75 nm cut-off, respectively. The secondary structure of peptides was analyzed using the DSSP program. Following a previous protocol^38^ of analyzing lipids in layers, we calculated the lateral diffusion coefficient (DL) for the first 10, 20 (excluding the first 10 lipids) and 64 lipids closest to the peptide in the monolayer in which the peptide was bound. 2D membrane thickness was obtained using the SuAVE analysis package^63^, which fits a rectangular grid mesh based on the location of membrane atoms (i.e. phosphorus atoms) and then calculates the average thickness by computing the distance between the upper and lower grids. Peptide density and the percentage of peptide volume inserted (Vpept) was calculated using the *s_dens* command from SuAVE.

#### Coarse-graining simulations and analysis

For coarse-graining simulations, we used the Martini3^64–66^ force field. All peptide atomistic structures were converted to coarse-grained structures with the *Martinize2* script^67^, including the -scfix flag and assigning alpha-helical secondary structure for BP100, Decoralin, and Temporin-L. For NK-1, the first four residues (Arg-Pro-Lys-Pro) were assigned as a random coil due to the presence of proline residues and the remaining aminoacids as alpha helical. Elastic network models and virtual G*ō* sites were not applied.

To get a more direct comparison between experimental and theoretical data and obtain mechanistic insights at a molecular level, peptide/vesicle systems were also prepared.

The initial vesicle structures were obtained from the CHARMM-GUI webserver^68^, using *Vesicle Builder* in *Martini* *Maker*^69^*.* Although experimental LUVs are close to 100 nm in diameter, we chose to simulate vesicles of approximately 20 nm in diameter-size which retain the structural similarity of larger LUVs while reducing computational cost and allowing proper sampling time. In order to compare directly with LUV leakage experiments, we used mixed vesicles of POPC and POPG at equal proportion (1:1 mol/mol), with symmetrical composition between leaflets (1772 and 1106 lipids in the outer and inner leaflets, respectively), and pure POPC vesicles (1701 and 1057 lipids in the outer and inner leaflets, respectively).

Vesicles were hydrated with CG water in a 30 × 30 × 30 nm simulation box and neutralized with counter-ions (Fig. 1F).

The vesicles obtained from CHARMM-GUI contained six water pores with a radius of 20 Å, allowing for lipid flip-flop and free movement of water molecules to equilibrate the interior and exterior compartments. Equilibration runs of vesicle-only systems were performed in 5 steps, with decreasing water pore diameter and increasing simulation time-steps (see Table S2 for detailed information). After equilibration steps, production runs of the pure lipid vesicles were performed for 10 µs and used as controls.

To explore peptide effects on vesicles at increasing peptide concentration, we built 5 systems by adding peptides into pre-equilibrated vesicle-only systems, reaching a peptide:lipid (P/L) ratio of 0.01 (lowest), 0.05 (low), 0.10 (medium), 0.20 (high), and 0.30 (highest). Peptides were randomly positioned in the extra-vesicular solution, using the *gmx insert-*molecules command. Table 1 shows all the simulation set-ups used in this work. BP100 was used as the representative SCHAMP. Thus, all vesicle simulations set-ups shown in Table 1 were performed with BP100. Following the insights obtained from the simulations of BP100 in POPC vesicles, we chose to simulate only POPC:POPG vesicles set-ups for the remaining three SCHAMPs.

**Table 1:** All-atom (AA) and coarse-graining (CG) simulation set-ups utilized in this work. For Decoralin, NK-1, and Temporin-L all set-ups were performed except CG simulations with POPC vesicles.

	Forcefield	System	# Peptides	Pressure (bar)	Temperature (K)	Time **(**μs)
Bilayer Membrane (AA simulations)	Amberff99sb-ildn-NMR (peptides) SLipids (lipids)	POPC:POPG (1:1) 64 lipids per leaflet	1	1	323	2
Vesicle (CG simulations)	Martini3	POPC:POPG (1:1) (Outer: 1722 lipids) (Inner: 1106 lipids)	28		303	10
			141			10
			283			50
			565			50
			848			50
		POPC (Outer: 1701 lipids) (Inner: 1057 lipids)	28			10
			138			10
			276			10
			551			10
			827			10

For peptide-vesicle CG simulations, energy minimization was performed using the steepest descent method for 10,000 steps. Systems were then equilibrated for 10 ns with a 10 fs timestep. For production runs we used a 20 fs time step and ran each system for 10 µs. Simulations were extended to 50 µs when needed. Pressure coupling was achieved using the Berendsen barostat in the equilibration step and for MD production runs we used the Parrinello-Rahman^62^ barostat using the same pressure coupling parameters (isotropic at 1 bar, coupling constant of 12.0 ps^−1^, and compressibility at 3 × 10^−4^). For both equilibration and production runs, we used the V-rescale^61^ thermostat at 303 K using a coupling constant of 1.0 ps^−1^. For all simulations, including minimization, equilibration and production runs, the neighbor list was updated using the Verlet search algorithm with a van der Waals interaction cut-off of 1.1 nm^70^. The reaction-field method was used to treat Coulomb interactions using a 1.1 nm cut-off with a dielectric constant of the reaction field set to infinity^70^. Vesicle structural properties were analyzed using the SuAVE package for closed surfaces^71^.

## Results

### All-atom simulations of single peptides on anionic membranes

**Figure 2:**
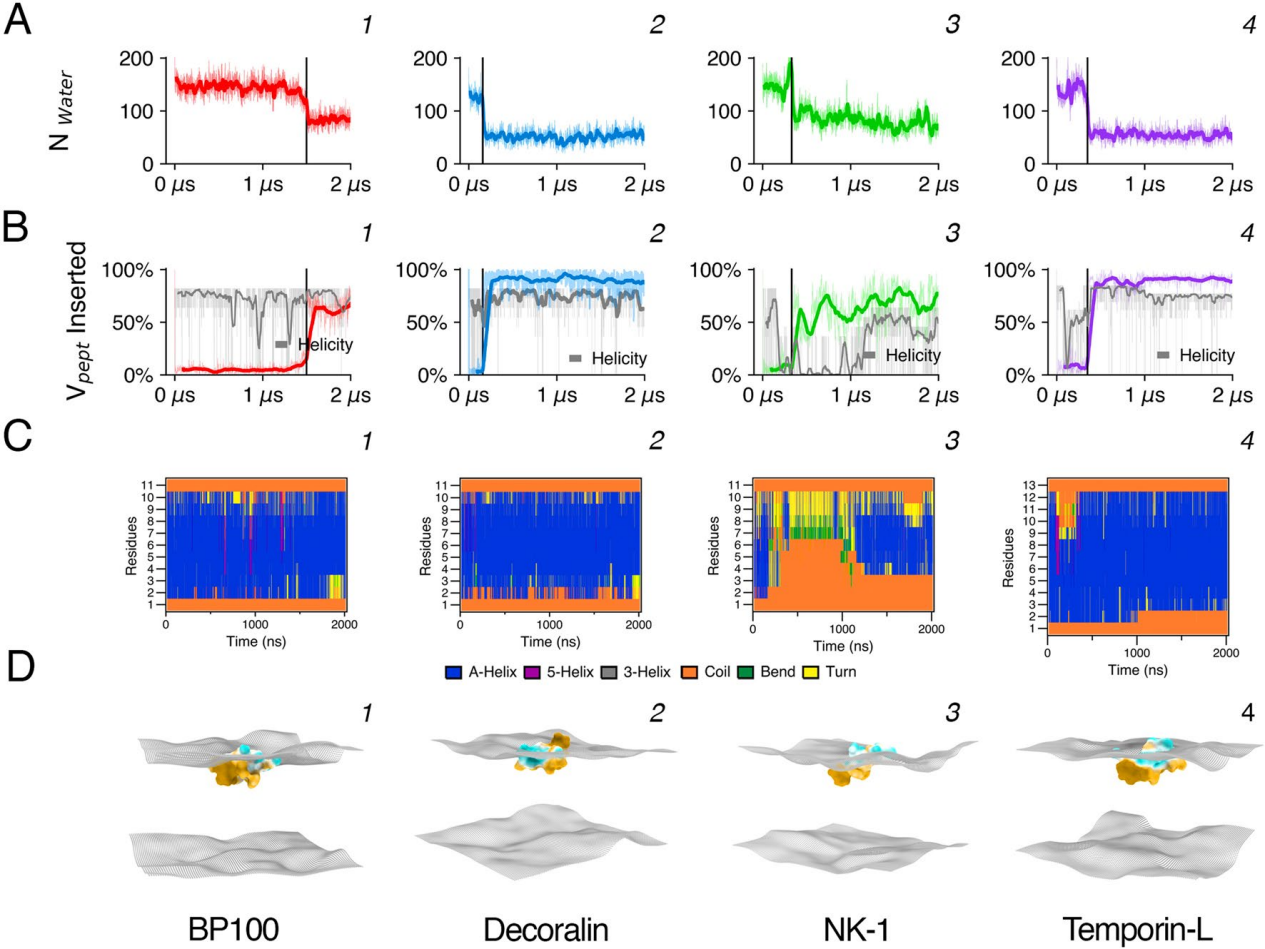
Peptide hydration, insertion, and structural analysis from atomistic simulations. For peptide hydration (**A**), waters surrounding the peptide within a 0.5 nm cut-off (Nwater) were computed for BP100 (column 1), Decoralin (column 2), NK-1 (column 3), and Temporin-L (column 4) throughout the simulations. Peptide flip occurred for all peptide:membrane simulations, at ~ 1.5 μs, ~ 0.16 μs, ~ 0.65 μs, and ~ 0.35 μs for BP100, Decoralin, NK-1 and Temporin-L, respectively. Black vertical lines in column 1 indicate the approximate time when peptide flip was observed. Peptide flip is accompanied by peptide steep dehydration (**A**) and insertion (**B**, Vpep Inserted). In (**C**), DSSP analysis show alpha helical structure (blue) favors peptide flip. Snapshots of the last frame of each simulation are shown in (**D**). Peptides are represented with their hydrophobicity surface, with hydrophobic areas in beige and hydrophilic regions in cyan. Surfaces were generated using SuAVE, using the phosphorus positions.

**Figure 3:**
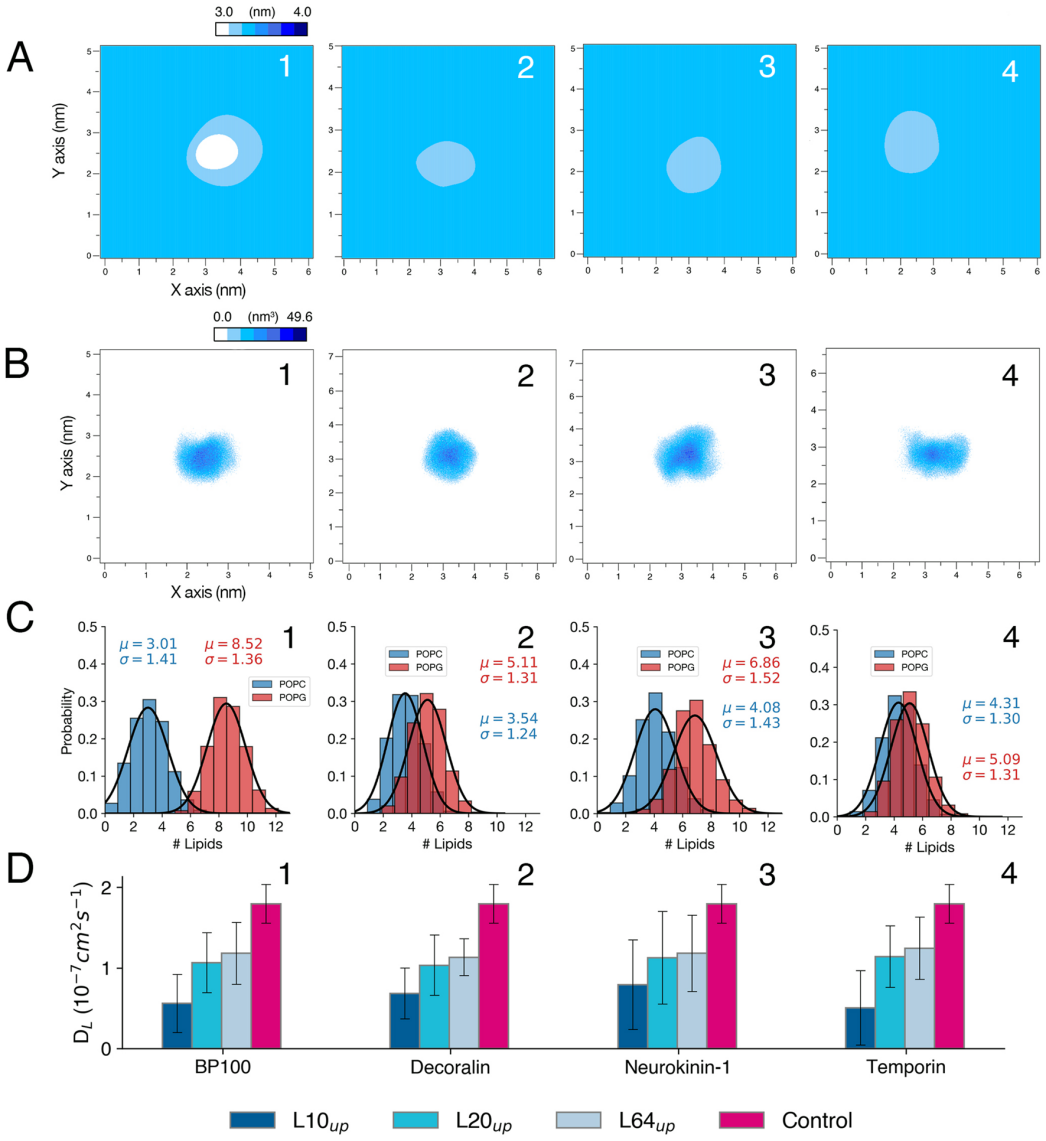
Antimicrobial peptides decrease membrane thickness and lipid diffusion locally. (**A**) and (**B**) show respectively 2D thickness mappings and peptide density for BP100 (column 1), Decoralin (column 2), NK-1 (column 3), and Temporin-L (column 4) in POPC:POPG membranes. The same enumeration is applied for other data. The probability distribution of POPC and POPG lipids in contact with peptides with their respective average and standard deviation values are shown in (**C**). Lipid diffusion (**D**) was computed for all peptides selecting the first 10, 20 neighboring lipids and for the whole monolayer (upper). Control values are reported for comparison.

To facilitate the comparison of peptide/membrane interactions, the results shown in Figs. 2 and 3 use a nomenclature identifying the property being analyzed by referring to the respective rows (A–D) and column (c). For example, “Fig. 2C, c1” refers to the DSSP analysis of BP100 during the simulated time (See below in the legend of Fig. 2).

The SLipids forcefield was previously validated^37^ and for the *ff99sb-ildn-NMR* forcefield, we validated it by simulating all 4 peptides in water and in membranes and comparing their secondary structure profile with structural experimental data^21,40,41,44^ (Fig. S1; Fig. 2, c3). As expected^52^, the peptides showed an over-helical behavior in water (Fig. S1), and the forcefield reproduced experimental structural properties in membranes (Fig. 2, column 3).

The four peptides chosen for this study—BP100, Decoralin, NK-1, and Temporin-L—fold into an alpha-helix on negatively charged membranes but do not have any structure in water^21,40,41,44^. Secondary structure analysis wasperformed using the DSSP algorithm (Fig. 2C), implemented in GROMACS. In all of the peptide/POPC:POPG atomistic simulations, we found a predominance of alpha-helical structures, except NK-1. On average, we found 75% of alpha-helix for BP100, 74% for Decoralin, 36% for NK-1, and 75% for Temporin-L throughout the simulations.

Similarly to our previous findings with saturated phosphatidylglycerol (DPPG) membranes, BP100 alpha helix was stable in POPC:POPG membrane (Fig. 2C, c1). The amino acid composition and helical projection of Decoralin is similar to BP100 (Fig. 1A, B) and, as expected, its secondary structure was predominantly alpha helical and stable along the simulation (Fig. 2C, c2). Though NK-1 has prolines residues in its N-terminus (Arg-Pro-Lys-Pro), we found no polyproline helix when analyzing the ψ and φ backbone dihedral angles of NK-1 first 4 residues in a Ramachandran plot (Fig. S2). As expected, the residues closer to the N-terminus remained unstructured due to the presence of Prolines (Pro2, Pro4) and when inserted into the membrane, NK-1 recovered its alpha-helix content in the half part closer to its C-terminus (Gln5, Gln6, Phe7, Phe8, Gln9, Leu10) (Fig. 2C, c3). As for Temporin-L, our secondary structure findings are in line with experimental CD^44^ and atomistic simulation data^47^ of Temporin-L in POPE/POPG and POPG membranes, with an overall alpha-helical structure.

Peptide flip is related with the peptide structure on membranes^37^. When folded into an alpha-helix, most SCHAMPs have a clear separation of hydrophilic and hydrophobic moieties, providing its amphiphilic character and favoring peptide rotation and insertion into the membrane. Trajectory analysis revealed peptide flip occurred for all the peptides investigated here (Fig. 2D). In this set of simulations the flipping times were ~ 1.5 μs (BP100), ~ 0.16 μs (Decoralin), ~ 0.65 μs (NK-1), and ~ 0.35 μs (Temporin-L) in POPC:POPG membranes (Fig. 2D). Similarly to our previous results with BP100 in DPPGbilayers^37^, peptides approached the POPC:POPG membrane with their charged residues portion, showing that the initial interaction is determined by electrostatic interactions. In this initial electrostatic-determined interaction the helical peptides were oriented parallel to the membrane surface. After variable times the peptides rotated and their hydrophobic residues were inserted into the membrane core, burying the peptides inside the bilayer (Fig. 2D). Peptide dehydration was coupled with peptide flip (Fig. 2A). The number of water molecules in the first hydration shell (r = 0.5 nm) decreased *ca.* 50% after the flip^37^ (Fig. 2A). Using the SuAVE membrane analysis package^63^, we generated surface grids based on the membrane phosphorus atom positions and calculated the percentage of peptide volume inserted (Vpept) between the grids (Fig. 2B). Vpept remained constant after the flip (Fig. 2B), showing the flipped-inserted state was stable during our simulations.

We also analyzed the effect of peptide binding on membrane properties. Previous simulation^38^ and experimental^18,72^ data shows that BP100 causes local thinning, evidenced by the matching of the concavity observed in 2D mappings with the peptide locations (Fig. 3A, B). Selectively calculating lipid lateral diffusion (DL) for the first neighboring 10, 20, and 64 lipids on each monolayer, we found that the 10 closest lipids were those most affected by the peptide binding, confirming its local activity (Fig. 3D). The DL for the 10 lipids neighboring the peptide decreased 69% (BP100), 62% (Decoralin), 55% (NK-1), and 72% (Temporin-L), respectively.

The number of lipids surrounding the peptides, computed using a 0.75 nm cut-off, shows the probability distribution of lipids in contact with the peptides (Fig. 3C). All peptides seem to cluster negatively charged phospholipids, as previously observed for BP100 and other AMPs in PG containing bilayers^37,73^. Clustering of POPG was not a consequence of peptide flip as it can be observed even before the occurrence of the flip. Rather, it seems to be related to the relative number of positively charged residues in the antimicrobial peptide amino acid composition and its overall hydrophobicity <H> (Table S1). Peptides with lower <H> and/or with higher percentage of positively charged residues in its sequence segregated larger numbers of POPG lipids, such as BP100 (+ 6, <H>  = 0.427) and NK-1 (+ 3, <H>  = 0.501).

Conversely, both Decoralin and Temporin-L possess an overall + 3 charge, but their overall hydrophobicity is high (0.780 and 0.906, respectively) compared to BP100 and NK-1. The presence of hydrophobic residues would increase the number of non-polar contacts between peptide and membrane acyl chains regardless of the lipid charge while the cationic peptide residues would attract specifically anionic lipids into the peptide surroundings.

### Higher P/L ratios with coarse-graining simulations

In order to study the collective action of multiple peptides, we resorted to coarse-grain (CG) simulations. Enabled by the computational efficiency of CG models, instead of planar membrane we used small vesicles to better mimic typical experimental setups. First, we simulated BP100 in POPC and POPC:POPG (1:1 mol/mol) vesicles using five peptide to lipid ratios (P/L): 0.01, 0.05, 0.10, 0.20, and 0.30. In all CG simulations with vesicles, peptide flip was observed for bound BP100, for both POPC and POPC:POPG vesicles, in line with the all-atom results.

The average number of bound peptides to POPC vesicles reached a plateau around P/L = 0.10 (Fig. S3B). When only bound peptides are considered, the ratio is close to P_bound_/L = 0.05 (Fig. S4A). From this point, the vesicles surfaces were saturated with peptides and the remaining peptides stayed in solution. At P/L = 0.10, BP100 increased the overall vesicle volume by 1.2% and membrane thickness by 1.1%, but no shape change of the vesicle was evident in the simulations (Fig. S3 and Table S3).

**Figure 4:**
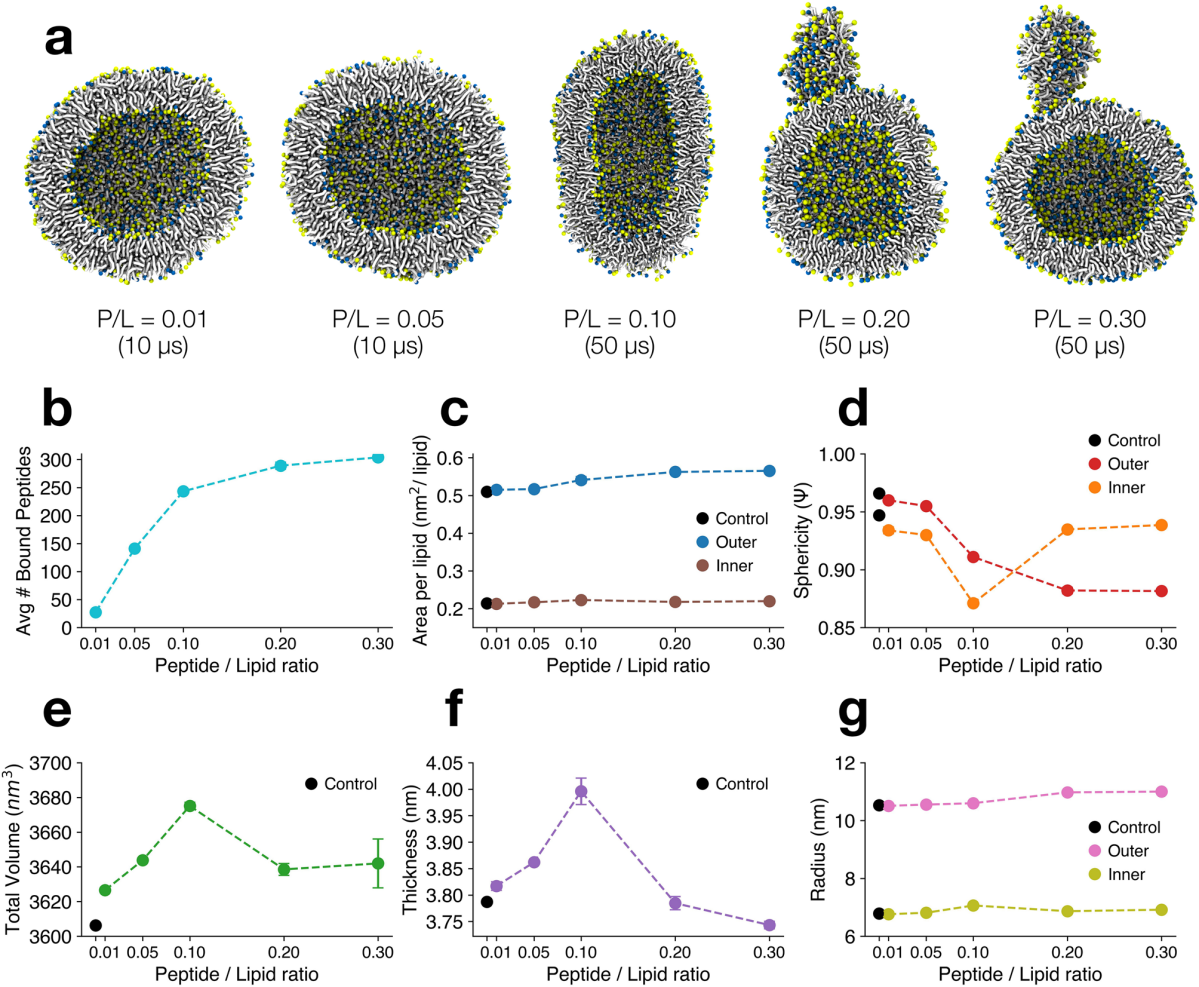
Antimicrobial BP100 induces protrusion formation in vesicles at high peptide:lipid ratios. (**a**) Cut-away cross-sectional last frame snapshots of POPC:POPG (1:1) vesicle/BP100 system at low (P/L = 0.01 and 0.05), medium (P/L = 0.10), and high (P/L = 0.20 and 0.30) concentrations. Peptides, water and ions are not shown for clarity. Obtained averaged peptide binding outcomes in vesicles according to the respective P/L are shown (**b**–**g**). Data points with no error bars had an error below 5%. Data points were obtained averaging the last 5 μs of each simulation. Errors are estimated by block averaging.

The simulations of negatively charged vesicles (POPC:POPG, at 1:1 mol/mol ratio) at low and medium P/L (0.01–0.10) reached equilibrium within 5 μs (Fig. S5). In contrast to the POPC vesicle system, the presence of negatively charged lipids caused virtually all peptides to bind (Figs. 4B, S4A) and remaining attached throughout the simulations (Fig. S5). Compared with a control POPC:POPG vesicle, low and medium P/L simulations showed a proportional increase of the area per lipid (APL), total vesicle volume (Vtotal), membrane thickness (DHH), and average radius (R) with peptide concentration (Fig. 4C, E–G, Table 2).

**Table 2:** Variation in percentage of vesicle structural properties compared to control.

System	APLouter (nm^2^/lipid)	APLinner (nm^2^/lipid)	Vtotal (nm^3^)	DHH (nm)	R (nm)	φ
Control	0.510	0.214	3606	3.79	10.5	0.966
P/L = 0.01	+ 1.0%	− 0.5%	+ 0.6%	+ 0.8%	− 0.2%	− 0.6%
P/L = 0.05	+ 1.4%	+ 1.9%	+ 1.0%	+ 2.0%	+ 0.2%	− 1.1%
P/L = 0.10	+ 6.1%	+ 3.2%	+ 1.9%	+ 5.5%	+ 0.6%	− 5.7%
P/L = 0.20	+ 9.8%	− 2.8%	+ 0.6%	− 0.2%	+ 3.8%	− 8.5%
P/L = 0.30	+ 10.4%	+ 1.0%	+ 0.8%	− 0.5%	+ 4.2%	− 8.7%

The overall vesicle shape can be monitored through sphericity (φ), an estimate of how closely the shape of an object resembles that of a perfect sphere. At low P/L, BP100 binding did not significantly alter φ compared to control (Fig. 4D, Table 2). However, at higher peptide ratios (P/L = 0.10), we calculated a -5.7% variation in φ compared to control (Fig. 4D, Table 2) and at the end of the simulation, the vesicle adopted an ellipsoidal shape, with highly curved edges (Fig. 4A, P/L = 0.10).

The vesicle remodeling into an ellipsoidal shape can be explained by the area increase upon peptide adsorption, effectively mimicking a hypertonic osmotic shock. Similar ellipsoidal structures have been observed both experimentally^74^ and theoretically^75^, upon the addition of membrane-binding agents. The proportional increase in membrane thickness from 0.01 to 0.1 P/L (Table 2) is in apparent disagreement with our previous atomistic simulations (Fig. 3A, ref.^38^) and experimental data^18,72^. However, differently from vesicle systems, planar membranes have intrinsically less curvature tension and thus the mechanism of action of SCHAMPs on planar and vesicular systems could differ. To prove this, we performed CG simulations of BP100 on planar POPC:POPG (50:50) bilayers at the same P/L ratios and observed a linear decrease in membrane thickness (Table S4).

At higher concentrations (P/L = 0.20 and 0.30), BP100 induced vesicle budding (Fig. 4A) and the average number of bound peptides reached a plateau at approximately P_bound_/L = 0.10. This value is similar to the saturation point found in titration experiments^76^ of BP100 in 2:1 POPC:POPG LUVs (P_bound_/L ≈ 0.12) and nearly double that observed in the POPC vesicle simulations at the same peptide concentration (Fig. S4A). We simulated the vesicles at both concentrations for 50 μs and vesicle budding was observed at ~ 10 μs and ~ 27 μs, respectively (Fig. S6). In both simulations, the same pattern as in P/L = 0.10 was observed. Initially, peptide binding alters the vesicle into an ellipsoidal shape as it increases the outer and inner APL (Fig. S6). Other vesicle properties increase proportionally, such as total vesicle volume, thickness and size (Fig. S6A, C).

Peptide-induced budding led to local detachment between acyl chains of the outer and inner monolayer lipids, thus allowing the inner leaflet to stabilize its APL and regaining its spherical shape, reaching control values (Fig. S6A, C). In contrast, in the outer leaflet, more peptides bound (Fig. S6A, B), increasing the outer APL, and thus growing the protrusion-body size along the simulation (Fig. S6). Concomitantly with budding, the average vesicle thickness decreased as fewer lipids on the outer leaflet were available to counter-part the inner vesicle lipids (Table 2, Fig. S6). Such thinning could make the peptide-enriched vesicles more prone to pore formation or further induce inner content leakage. No lipid flip-flop between outer and inner vesicle lipids nor full separation between vesicle and protruding buds were observed during our simulation time (Fig. S6B, D).

The appearance of a budding transition at higher P/L ratios can be explained by an increase in spontaneous membrane curvature. In addition to membrane expansion, the adsorption of peptides to the outer leaflet only causes an asymmetric stress (positive curvature), driving the bud formation in line with experimental and theoretical findings^74,76^.

Next, we also simulated Decoralin, NK-1, and Temporin-L interacting with POPC:POPG vesicles at the same P/L ratios (0.01, 0.05, 0.10, 0.20, and 0.30). Following our secondary structure data from atomistic simulateons (Fig. 2C) all peptides except NK-1 were assigned as full alpha-helical CG structure. As the first 4 residues of NK-1 had no secondary structure (Fig. 2C, c3), in this case only residues 5 to 11 were assigned as alpha-helix in our CG simulations.

**Figure 5:**
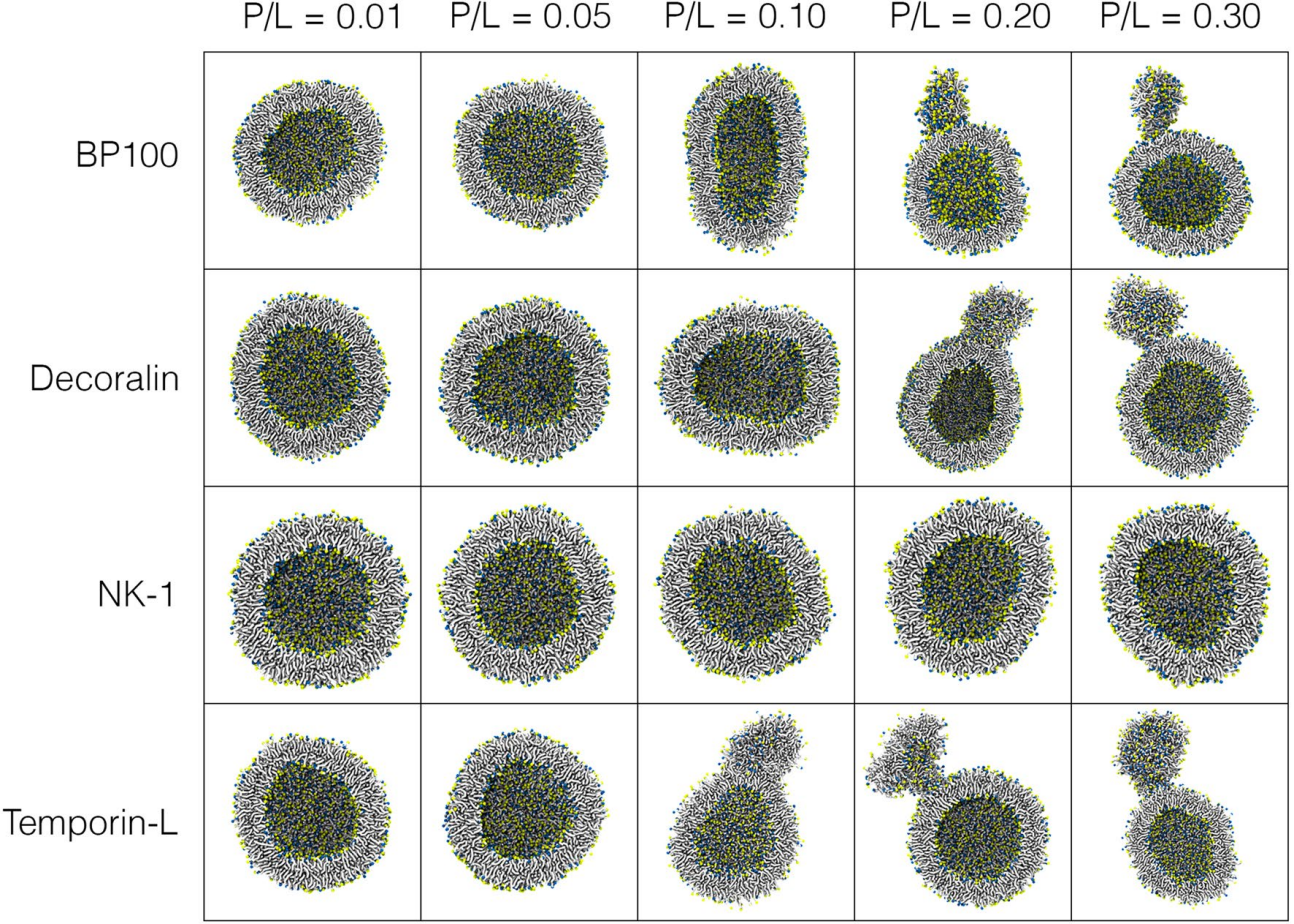
Short cationic alpha-helical peptides cause vesicle budding at high concentrations. Cut-away cross-sectional last frame snapshots of POPC:POPG (1:1) vesicles with BP100, Decoralin, NK-1, and Temporin-L simulations. Water, counter-ions, and peptides are not shown for clarity.

Such difference in secondary structure proved to be key in the outcomes of our peptide/vesicle CG simulations. NK-1 had no effect on the vesicles shape in all P/L ratios simulated (Fig. 5, NK-1). Like BP100 (Fig. 5, BP100), Decoralin peptides (Fig. 5, Decoralin) had little effect on POPC:POPG vesicles at low P/L (0.01and 0.05). At medium P/L (0.10), Decoralin altered the vesicle into an ellipsoidal shape. At high P/L (0.20 and 0.30), it created highly curved regions in the vesicle and eventually membrane budding was observed. Simulations with Temporin-L showed the same pattern except that membrane budding occurred from P/L = 0.10.

## Discussion

Overall, our simulation results suggest SCHAMPs act on model membranes via the carpet mechanism at high peptide:lipid ratios. Such a finding was suggested for BP100 experimentally^17,18,21,24,77^, but our simulation data show it can be expanded for other peptides that share similar folding and length. Our CG results using the latest version of Martini (3.0), are similar to those obtained by Woo et al., who first reported a budding effect on membranes by magainin-2, using Martini 2.2^78^. More recently, similar peptide-induced membrane budding was reported by Zhang et al.^79^, when simulating Temporin B and L on POPC:POPG (7:3) planar bilayers. Using the pSPICA CGforcefield, Miyazaki and Shinoda^80^ conducted melittin simulations on POPC planar membranes and vesicles and reported identical budding and lipid extraction at P/L ~ 0.10 (when considering melittin’s double length compared to SCHAMPs). The authors also described the alteration in the vesicle morphology into an ellipsoidal shape upon peptide binding, and further pore formation.

Our atomistic simulations showed all four peptides caused local membrane thinning and negative curvature (Fig. 3A). At the individual level, SCHAMPs bind and flip on the membrane, the peptide buries into the membrane and the hydrophobic facet can deeply insert into the membrane core and decrease the order parameter of lipid acyl chains, causing local membrane thinning^18,38,72^. It is probable that actual peptide insertion into the membrane does not follow a sequential progression, as observed in our simulations, involving peptide folding, flipping, and subsequent insertion into the core. Instead, it is more likely that all events occur simultaneously in a concerted fashion. Consequently, the concept of peptide flip should be regarded as a transition between the solvated peptide state to the bound peptide state. However, at higher P/L, the steady and continuous binding and flipping of SCHAMPs into the membrane creates an imbalance in the overall membrane volume and APL between the outer and inner leaflets. Such imbalance first promotes vesicle swelling, increasing outer and inner APL and membrane thickness, and thus, positive curvature is created (Table 2). When enough peptides are bound and highly curved regions are produced in vesicles (Fig. S6B, D), membrane budding starts. We calculated the average number of peptides bound to the membrane for BP100 in POPC and POPC:POPG membranes (Figs. 4B and S3B), and we found that they saturate at approximately P_bound_/L = 0.05 and 0.10, respectively. We observed that after budding, more peptides bound to the vesicle (Fig. S6A, D), increasing the outer APL and the protrusion size, while membrane thickness decreased.

Such outcome could lead to pore-formation or complete dissociation between the protrusion and vesicle. It is noteworthy that, due to computational restraints, our simulations focused on small vesicles (~ 20 nm in diameter). In larger vesicles, SCHAMPs possibly could induce more drastic outcomes, such as vesicle fission or inner content leakage through pore-formation.

The differences between the studied peptides in their binding and membrane outcomes can be explained with the “wedge” model^81^ (see reference for detailed description). In this model, SCHAMPs have a cross-sectional wedge shape, specific to their amino acid composition. Peptides featuring a wide polar face of charged residues and a narrow non-polar face are denominated as wedge-shaped peptides. SCHAMPs with a wider hydrophobic face and an apex consisting of a smaller cluster of cationic residues, are the inverted wedge-shaped peptides. At low P/L, wedge-shaped peptides can generate positive curvature while inverted wedge-shaped peptides produce negative curvature on membranes^82–84^. However, at high P/L, both types of peptides could generate positive curvature as bound peptide volume contribution increases.

Inverted wedge-shaped SCHAMPs, such as Temporin-L, are more hydrophobic than wedge-shape peptides, leading to higher percentage of bound peptides to the membranes even at lower and medium P/L. And thus, peptide-induced budding could occur even at P/L = 0.10 (Fig. 5, Temporin-L).

The number of non-polar amino acids with large side-chains seem to have a key role in SCHAMPs activity. Leucine, phenylalanine, tyrosine, and tryptophan are found in many AMPs^85^. In the case of Temporin-L, when substituting both Phe3 and Phe5 with leucines, the modified Temporin-L has no activity against *E. coli* and *P. aeruginosa*^86^. When folded into an alpha-helix, Temporin-L has a clear spatial segregation between hydrophobic and hydrophilic residues and the aromatic amino acids are concentrated in the hydrophobic portion (Fig. 1D). A similar behavior was reported in an alanine-scan study with BP100^77^. While substituting BP100 positively charged residues produced little to no effect in minimum inhibitory concentrations against Gram positive and negative bacteria, replacing hydrophobic residues with larger side groups with Alanine, such as Leucine, Phenylalanine, and Tyrosine, drastically reduced BP100 antimicrobial activity^77^.

NK-1 contains proline amino acids which destabilize alpha-helix folding on the water/membrane interface, and although NK-1 has 2 aromatic amino acids (Phe), the hydrophobic/hydrophilic facets are not clearly separated, decreasing its hydrophobic moment and consequently decreasing its affinity with the membrane and peptide flip occurrence. Decoralin and BP100 have similar features in terms of amino acid composition, folding behavior in water and membranes, and clear hydrophobic/hydrophilic facet separation when in alpha-helical conformation. Moreover, both peptides possess residues with medium to large side-chains in their hydrophobic moiety (BP100—Leu, Phe,Tyr; Decoralin—Leu, see Fig. 1A, B).

The wedge model, is a convenient way to characterize some of the peptide effects observed here. We recognize, however that this model should not be the only parameter when ranking suitable antimicrobial peptides for pharmaceutical use. Inverted wedge-shaped SCHAMPs are likely to be more toxic towards human cells, such as Temporin-L^42^, due to their higher affinity toward membranes.

Although our CG findings indicate SCHAMPs act via the carpet mechanism, one should be mindful of the limitations of AA and CG simulations. AMPs pore formation phenomena are in the microsecond scale^29,30^, and therefore longer AA simulations with increased SCHAMP concentrations could reveal a different outcome. Moreover, Martini overestimates the energy cost for pore formation compared to AA forcefields^87^, probably due to the implicit screening of charges in Martini^64^ and thus, the budding observed in our simulations could be the result of the current inability of Martini of simulating the formation of pores^35^. The size constraints of our Martini simulation set-ups may also present as a barrier to alternative ways of relieving bilayer mismatch tension, such as membrane buckling or tubulation. Finally, the role of peptide folding on the membrane interface is absent in Martini, in which peptide secondary structure is pre-determined and fixed into an alpha-helix^65^. Another interpretation of our results could be the possible occurrence of a simultaneous (with pore and budding formation) or a sequential membrane disruption mechanism, with budding as a short-lived intermediate state before pore formation.

In summary, our results suggest that BP100, Decoralin, NK-1, and Temporin-L share common mechanism steps when destabilizing anionic membranes. Our atomistic simulations revealed all 4 SCHAMPs flipped on anionic membranes at low peptide concentration, and were accompanied by peptide dehydration and insertion into the membrane core. All peptides perturbed the bilayers locally, with membrane thinning (Fig. 3A, B), anionic lipid aggregation (Fig. 3C) and decreasing lipid lateral diffusion (Fig. 3D). In our CG vesicle simulations with higher peptide concentrations (P/L = 0.20 and 0.30), full alpha-helical peptides (BP100, Decoralin, and Temporin-L) deformed vesicles and induced budding in highly curved regions of the vesicles. The inverted wedge mechanism coupled to the contributions of large hydrophobic amino acids can explain the budding in vesicles by SCHAMPs. Future work on SCHAMPs, such as with improved forcefield parameters, polarized water models, and even with pre-formed larger pores could also shed light on other alternative mechanisms of SCHAMPs.

### Supplementary Information

Supplementary Information.

## Data Availability

For the all-atom and coarse-grained Molecular Dynamics simulations, data (initial structures, cleaned trajectories, simulation parameters) are available through the openrepositoryZenodo [https://doi.org/10.5281/zenodo.8408508].
