# Supplementary material for: Vesicle protrusion induced by antimicrobial peptides suggests common carpet mechanism for short antimicrobial peptides
Source: Sci Rep. 2024 Apr 27;14:9701. doi: 10.1038/s41598-024-60601-w (PMC11055889; doi:10.1038/s41598-024-60601-w)
Supplement: Supplementary file 1 — Supplementary Information. [file 41598_2024_60601_MOESM1_ESM.docx]

### **Supplementary Material**

**Vesicle protrusion induced by antimicrobial peptides suggests common carpet mechanism for short antimicrobial peptides**

Peter Park^1,3^, Danilo K. Matsubara^1^, Domenico R. Barzotto^1^, Filipe S. Lima^2^, Hernan Chaimovich^1^, Siewert J. Marrink^3,*^, Iolanda M. Cuccovia^1,*^

^1^ Departamento de Bioquímica, Instituto de Química, Universidade de São Paulo, São Paulo, Brazil;

^2^ Departamento de Química Fundamental, Centro de Ciências Exatas e da Natureza, Universidade Federal de Pernambuco, Recife, Brazil.

^3^ Groningen Biomolecular Sciences and Biotechnology Institute (GBB), University of Groningen, 9747 AG Groningen, the Netherlands

|  | | **BP100** | **Decoralin** | **NK-1** | **Temporin** |
| --- | --- | --- | --- | --- | --- |
| Sequence | | KKLFKKILKYL | SLLSLIRKLIT | RPKPQQFFGLM | FVQWFSKFLGRIL |
| Aminoacids | | 11 | 11 | 11 | 13 |
| Overall charge | | +6 | +3 | +3 | +3 |
| <H> Hydrophobicity | | 0.427 | 0.780 | 0.501 | 0.906 |
| Aminoacids composition | Polar | 5 (45.45%) | 5 (27.78%) | 5 (27.78%) | 5 (27.78%) |
|  | Non-Polar | 6 (54.54%) | 6 (33.33%) | 6 (33.33%) | 8 (44.44%) |
|  | Charged | 5 (45.45%) | 3 (27.27%) | 2 (18.18%) | 3 (27.27%) |
|  | Aromatic | 2 (18.18%) | 0 (0%) | 2 (18.18%) | 4 (30.76%) |

**Table S1:** Structural properties of the antimicrobial peptides studied. The hydrophobicity <H> was calculated using the Fauchere-Pliska scale^1^.

|  | **Eq 1** | **Eq 2** | **Eq 3** | **Eq 4** | **Eq 5** | **Production** |
| --- | --- | --- | --- | --- | --- | --- |
| Water pore size (Å) | 20 | 15 | 10 | 5 | 2 | 0 |
| Time step (fs) | 2 | 5 | 10 | 15 | 20 | 20 |
| Time (ns) | 100 | 25 | 25 | 25 | 25 | 10000 |
| Barostat | Berendsen | | | | | Parrinello-Rahman |
|  | 1 bar  isotropic  𝜏_p_= 5.0 ps^-1^  *β* = 4.5e^-5^ | | | | | 1 bar  isotropic  𝜏_p_= 12.0 ps^-1^  *β* = 4.5e^-5^ |
| Thermostat | V-rescale (𝜏_t_=1.0, T=303.15 K) | | | | | |

**Table S2:** Vesicle-only system equilibration and production information. *β* is compressibility, 𝜏_p_ is the pressure coupling constant, and 𝜏_t_ is the temperature coupling constant

## Secondary Structure in Water

**
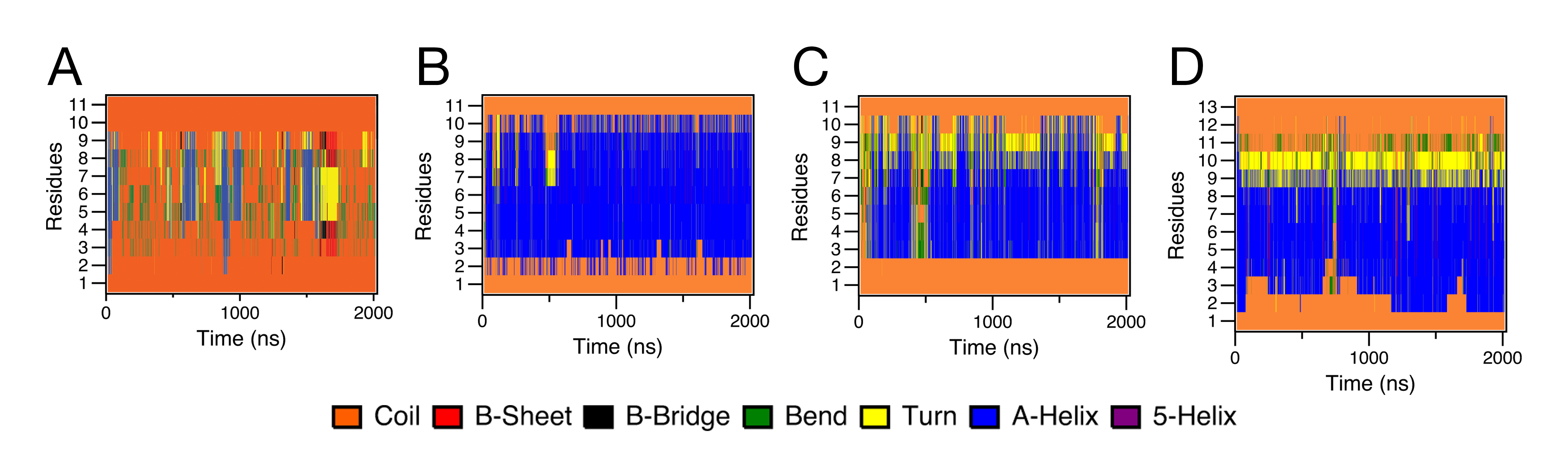
**

**Figure S1:** Secondary structure analysis (DSSP) of peptides in water from atomistic

simulations. BP100 (A), Decoralin (B), NK-1 (C), and Temporin-L (D). The table below shows the respective percentage of helicity obtained from the DSSP analysis.

| BP100 | Decoralin | Neurokinin-1 | Temporin |
| --- | --- | --- | --- |
| Helicity (%) | | | |
| 9 | 65 | 49 | 49 |
| Random Coil (%) | | | |
| 67 | 29 | 36 | 36 |


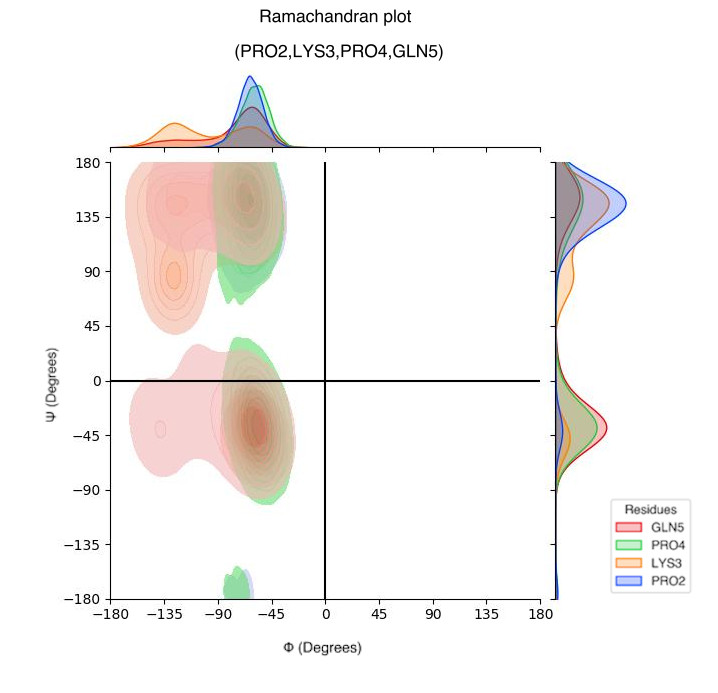


**Figure S2:** Ramachandran plot and respective histograms for NK-1 N-terminus region from the atomistic simulation in POPC/POPG membrane. The plot reveals the region has higher flexibity, as in a random coil conformation, which means residues can occupy randomly different regions of the graph at different times^2^, with some restrictions due to the more rigid nature of proline, given that it can only bend at a range of roughly -90 to -45 degrees along the ɸ axis^3^. For example, GLN5 occupies regions typically associated with beta sheets, PPII helices and alpha helices^4^.

**Figure S3**: **Simulations of BP100 in POPC vesicles.** (a) Cut-away cross-sectional last frame snapshots of POPC vesicle/BP100 system at low (P/L = 0.01 and 0.05), medium (P/L = 0.10), and high (P/L = 0.20 and 0.30) concentrations. Peptides, water and ions are not shown for clarity. Obtained averaged peptide binding outcomes in vesicles according to the respective P/L are presented (b-g). Data points with no error bars had an error below 5%. Data points and error bars were obtained averaging the last 5μ of each simulation.


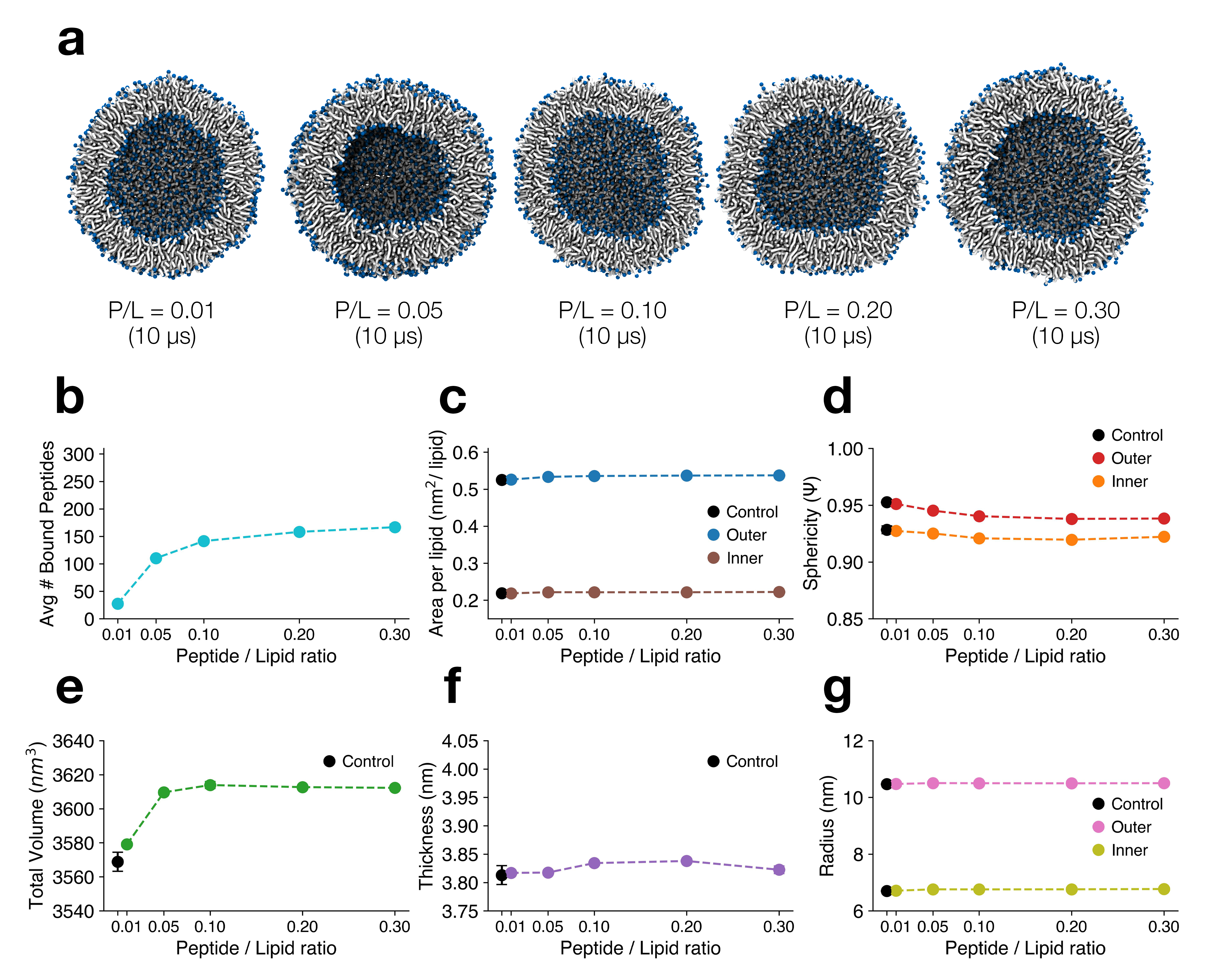


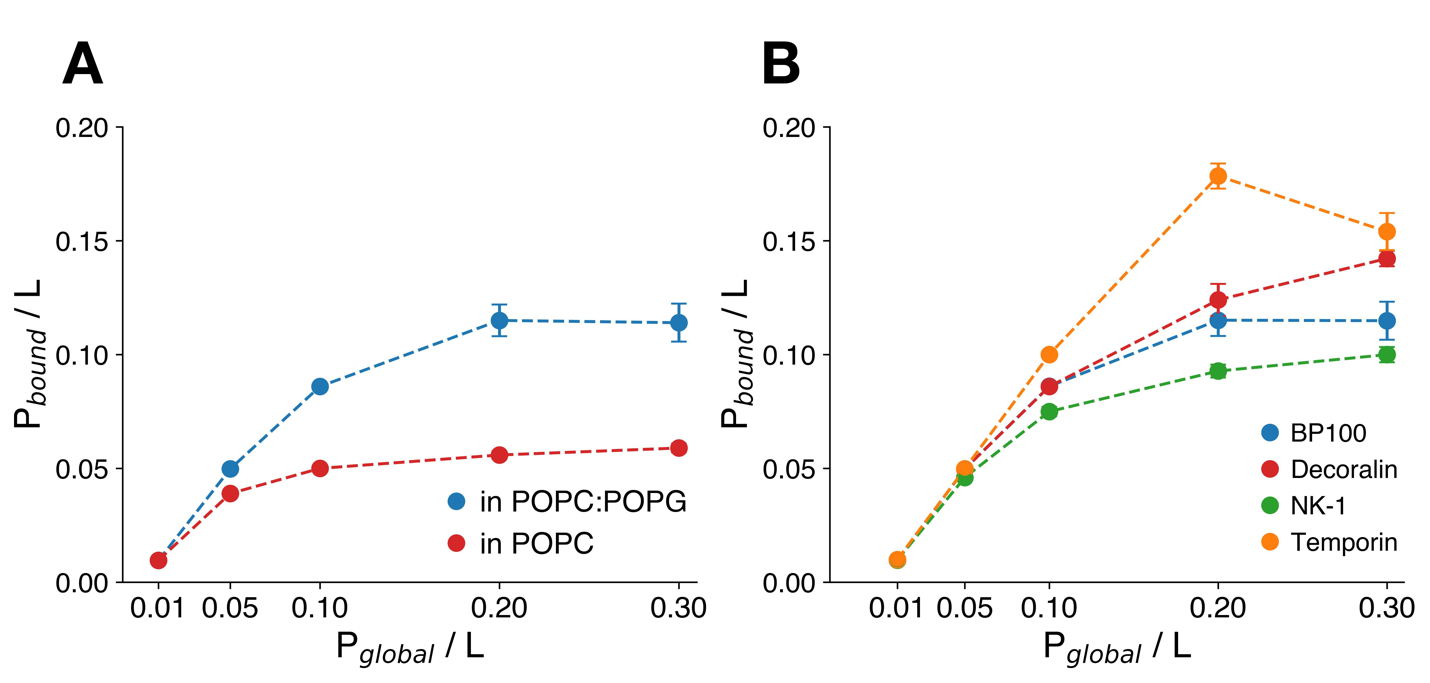


**Figure S4**: **Comparison of the total number of peptides bound to vesicles with the overall number.** In (a), we show the effective bound P_bound_/L for BP100 in POPC and in POPC:POPG vesicle simulations. P_bound_ is the average number of peptides bound to the membrane, L is the number of lipids, and P_global_ is the global number of peptides. In (B), we present the equivalent data for all SCHAMPs simulations in POPC:POPG (1:1 mol:mol) vesicles. Data points with no error bars had an error below 5%. Data points and error bars were obtained averaging the last 5μ of each simulation.

| System | APL_outer_  (nm^2^/lipid) | APL_inner_  (nm^2^/lipid) | V_total_  (nm^3^) | D_HH_  (nm) | R  (nm) | φ |
| --- | --- | --- | --- | --- | --- | --- |
| Control | 0.525 | 0.219 | 3568 | 3.81 | 10.4 | 0.952 |
| P/L = 0.01 | +0.19% | -0.17% | +0.29% | -0.17% | +0.07% | -0.15% |
| P/L = 0.05 | +1.54% | +1.24% | +1.14% | +1.24% | +0.37% | -0.78% |
| P/L = 0.10 | +1.99% | +1.16% | +1.26% | +1.16% | +0.32% | -1.29% |
| P/L = 0.20 | +2.20% | +1.18% | +1.23% | +1.18% | +0.30% | -1.54% |
| P/L = 0.30 | +2.32% | +1.52% | +1.22% | +1.52% | +0.34% | -1.50% |
| **Table S3: Summary of vesicle structural properties from BP100 in POPC vesicle simulations.** Variation in percentage of POPC vesicle structural properties compared to control. | | | | | | |


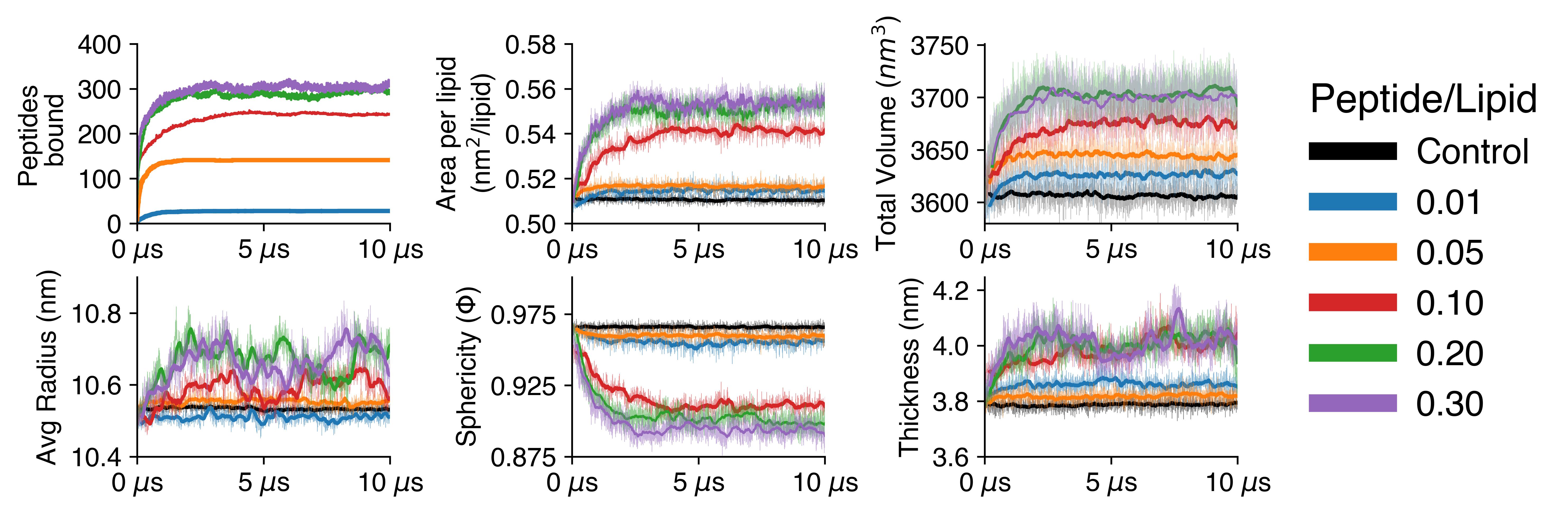


**Figure S5:** Temporal analysis of vesicle structural properties from BP100 in POPC:POPG (50:50) vesicle simulations.

| **System** | **D_HH_ (nm)** |
| --- | --- |
| Control | 3.924 |
| P/L = 0.01 | -0.71% |
| P/L = 0.05 | -3.47% |
| P/L = 0.10 | -6.15% |
| P/L = 0.20 | -6.34% |
| P/L = 0.30 | -6.50% |

**Table S4: Membrane thickness from BP100 in POPC:POPG (1:1 mol/mol) planar membrane simulations (288 lipids in each monolayer).** Variation in percentage of POPC:POPG membrane thickness compared to control. Estimated errors by block averaging were below 5%.


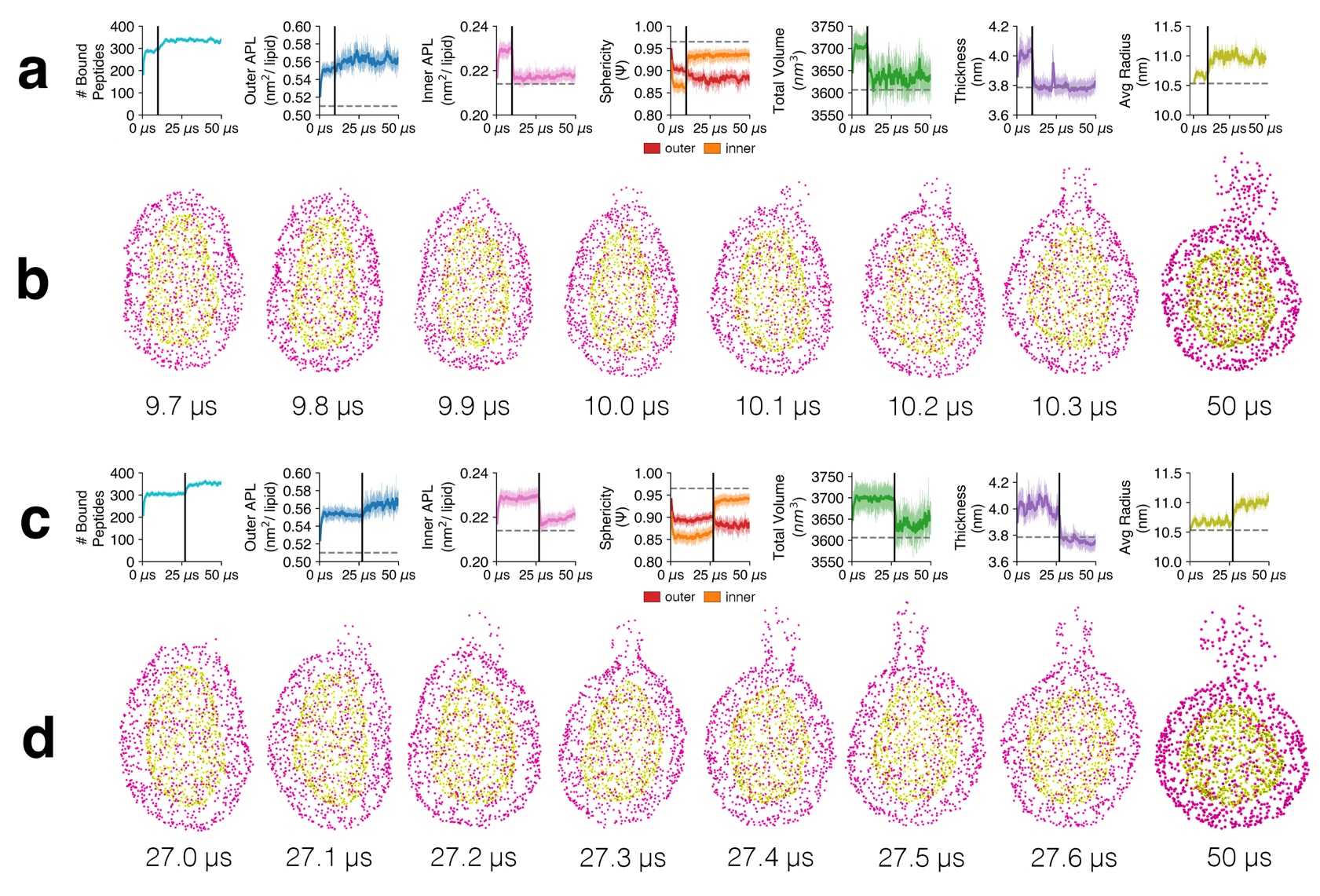


**Figure S6: Antimicrobial peptide BP100 induces vesicle budding at high concentrations.** (A) and (C) show computed properties for POPC:POPG vesicles throughout BP100/vesicle simulation at P/L = 0.20 and P/L = 0.30, respectively. We show the number of bound peptides, outer and inner area per lipid, outer leaflet sphericity, total vesicle volume, membrane thickness and average radius. Black vertical lines indicate the approximate time when budding started. (B) and (D) show snapshots of the vesicles of both simulations during membrane protrusion. Only phosphorus atoms are shown and are colored in magenta (outer) and yellow (inner).

.

**References**

1. Fauchere, J.-L. & Pliska, V. Hydrophobic parameters of pi amino-acid side chains from the partitioning of N-acetyl-amino-acid amides. *Eur. 1. Med. Chem. Chim. Ther.* **18,** 369-375 (1983).
2. Schweitzer-Stenner, R. (2012). Conformational propensities and residual structures in unfolded peptides and proteins. In *Molecular BioSystems* (Vol. 8, Issue 1, pp. 122–133). <https://doi.org/10.1039/c1mb05225j>
3. Adzhubei, A. A., & Sternberg, M. J. E. (1993). Left-handed Polyproline II Helices Commonly Occur in Globular Proteins. *Journal of Molecular Biology*, *229*(2), 472–493. <https://doi.org/10.1006/jmbi.1993.1047>
4. Hollingsworth, S. A., & Karplus, P. A. (2010). A fresh look at the Ramachandran plot and the occurrence of standard structures in proteins. In *Biomolecular Concepts* (Vol. 1, Issues 3–4, pp. 271–283). De Gruyter Mouton. <https://doi.org/10.1515/bmc.2010.022>
